# Action Prediction in Younger versus Older Adults: Neural Correlates of Motor Familiarity

**DOI:** 10.1371/journal.pone.0064195

**Published:** 2013-05-21

**Authors:** Nadine Diersch, Karsten Mueller, Emily S. Cross, Waltraud Stadler, Martina Rieger, Simone Schütz-Bosbach

**Affiliations:** 1 Max Planck Institute for Human Cognitive and Brain Sciences, Leipzig, Germany; 2 MaxNetAging Research School, Max Planck Institute for Demographic Research, Rostock, Germany; 3 Wales Institute for Cognitive Neuroscience, School of Psychology, Bangor University, Gwynedd, United Kingdom; 4 Behavioral Science Institute, Donders Institute for Brain, Cognition and Behaviour, Radboud University Nijmegen, Nijmegen, The Netherlands; 5 Movement Science Unit, Faculty for Sports and Health Science, Technical University Munich, Munich, Germany; 6 Institute for Psychology, Medical Sciences and Management Department, UMIT, The Health and Life Sciences University, Hall/Tyrol, Austria; University of Bologna, Italy

## Abstract

Generating predictions during action observation is essential for efficient navigation through our social environment. With age, the sensitivity in action prediction declines. In younger adults, the action observation network (AON), consisting of premotor, parietal and occipitotemporal cortices, has been implicated in transforming executed and observed actions into a common code. Much less is known about age-related changes in the neural representation of observed actions. Using fMRI, the present study measured brain activity in younger and older adults during the prediction of temporarily occluded actions (figure skating elements and simple movement exercises). All participants were highly familiar with the movement exercises whereas only some participants were experienced figure skaters. With respect to the AON, the results confirm that this network was preferentially engaged for the more familiar movement exercises. Compared to younger adults, older adults recruited visual regions to perform the task and, additionally, the hippocampus and caudate when the observed actions were familiar to them. Thus, instead of effectively exploiting the sensorimotor matching properties of the AON, older adults seemed to rely predominantly on the visual dynamics of the observed actions to perform the task. Our data further suggest that the caudate played an important role during the prediction of the less familiar figure skating elements in better-performing groups. Together, these findings show that action prediction engages a distributed network in the brain, which is modulated by the content of the observed actions and the age and experience of the observer.

## Introduction

As humans, our ability to successfully navigate through our social environment and interact with others is critical for survival. It has been argued that instead of just passively relying on sensory input during the observation of others' actions, we also generate internal predictions on what we see [1]–[5]. This enables us to adapt and respond more quickly and efficiently to changes in the environment. The ability to create these action predictions is thought to be based on a shared representation between executed and observed actions [6], [7].

So far, research on the prediction of observed actions focused mainly on younger age groups while neglecting changes in these processes over the lifespan. Evidence suggests that there might be a specific decline in how older adults anticipate observed actions, possibly due to less precise internal action representations (e.g., [8]–[13]). In a recent behavioral study, we showed that older adults predicted the time course of different action sequences less precisely than younger adults [8]. Although the timing in prediction was not systematically biased in older compared to younger adults (i.e., generally slower or faster), older adults did not seem to represent the observed actions in sufficient temporal detail in order to predict their exact time-course. This suggests that age-related differences in prediction performance are unlikely to be accounted for by general changes in time perception (cf., [14]). Our results rather imply an age-related decline in how observed actions are internally mapped onto one's own action representations. We further demonstrated that sensorimotor experience with observed actions resulted in a better prediction performance for domain-specific actions in both older and younger experts compared to non-experts. However, how the process of action prediction is implemented in the aging brain depending on the degree of motor familiarity with the observed actions remains an open question.

In the light of a demographic change visible in many countries with lower fertility rates and rising life expectancies, a better understanding of age-related changes in these vital abilities together with its neural basis is essential in improving skill learning and skill maintenance in older adults (cf., [15]). It is well known that the aging brain is subject to substantial changes at the structural as well as functional level [16]–[18]. In addition, older adults exhibit different task-related activation patterns compared to younger adults while performing the same task that could reflect neural dedifferentiation and/or compensation [19]–[21]. According to the scaffolding theory of aging and cognition (STAC), aging can be viewed as a (intrinsic) challenge to the human brain just as unfamiliar tasks pose a (extrinsic) challenge to a younger brain [22]. Both types of challenges result in a functional reorganization (i.e., neural scaffolding) to maintain or improve task performance. Whereas neural recruitment patterns in younger adults become increasingly specialized with training, older adults may recruit additional regions already during the performance of familiar tasks to compensate for noisy and/or inefficient neural processing. However, even in older age groups these neural recruitment patterns are amenable to training [23]. In sensory as well as motor processing, age-related differences in neural activity together with altered functional connectivity have been demonstrated by a number of studies, possibly reflecting less specific neural representations in action *and* perception with advancing age (e.g., [24]–[31]).

In younger adults, neuroimaging studies on action observation identified regions in the premotor and inferior parietal cortex that are similarly activated during action execution [32]–[35]. A network comprising these sensorimotor regions as well as occipitotemporal regions implicated in biological motion processing has been referred to as the *action observation network* (AON; [36]). By transforming executed and observed actions into a common code, the AON might serve as the neural substrate for the ability to predict the actions of others. According to the predictive coding account, the different regions of the AON are reciprocally connected and an actual representation of the observed action is compared to a predicted representation at each level of the cortical hierarchy [37]–[39]. This comparison generates a prediction error, which is back propagated through the cortical hierarchy to update the internal action representation and minimize the prediction error.

Consequently, shared representations between action and perception and their neural basis are assumed to be established through sensorimotor experience that strengthens the connectivity between relevant areas [40]–[42]. Studies on skilled motor performance frequently demonstrate superior prediction abilities in experts when they observe actions from their domain of expertise whereas non-experts rather rely on the visual dynamics of the observed actions resulting in a less efficient anticipation performance (e.g., [43]–[46]). Not surprisingly, AON activity has been shown to be modulated by the sensorimotor experience of the observer. The majority of studies investigating this issue has found increased activity in these regions during the observation of familiar actions as compared to actions that are not in the motor repertoire of the observer (e.g., [47]–[51]). Observers that are not familiar with the shown actions, in contrast, seem to recruit additional regions beyond the AON, for example, in visual cortices to perform these kinds of tasks (e.g., [52], [53]). In addition, recent evidence indicates that specific task requirements and stimulus characteristics might also result in the activation of regions that are not typically considered to be part of the AON during action observation [54]–[56]. For example, Schiffer and Schubotz [55] showed that prediction errors during action observation in ambiguous contexts are coded within a sub-region of the basal ganglia, the caudate nucleus. They suggested that the caudate might trigger the updating of the respective internal action representation if the sensory input violates the initial prediction.

To the best of our knowledge, there are only very few studies so far that examined age-related differences in the neural representation of observed actions. By using transcranial magnetic stimulation (TMS), Léonard and Tremblay [57] showed that corticomotor facilitation in relevant muscles is less specialized in older compared to younger adults during action observation, imitation, and imagery. In addition, Nedelko et al. [58] did not report age-related activation differences in the premotor and inferior parietal regions of the AON during observation and imagery of simple goal directed actions. The authors concluded that activity in these regions is age-independent. However, older adults recruited additional regions in the superior parietal and occipital cortices compared to younger adults, which might indicate a different processing of the observed actions (i.e., neural scaffolding). Yet, it remains unclear to what extent the prediction of an observed action is linked to similar changes at the neural level in older adults and whether they are modulated by the degree of motor familiarity in the aging observer.

By using fMRI, the present study examined the underlying neural activation patterns in younger and older adults during the prediction of action sequences that varied in their degree of motor familiarity (classical figure skating elements and simple movement exercises). All of the participants were highly familiar with the movement exercises, whereas only some of the younger and older adults possessed sensorimotor experience in figure skating. During fMRI scanning, participants were required to judge the temporal coherence of the observed action sequences that were partly occluded at critical time points and whose continuation afterwards was temporarily manipulated. Brain activity was examined as a function of observed action category and continuation after occlusion collapsed across the whole sample as well as a function of age group while controlling for the effects of sensorimotor experience in figure skating. In addition, brain activity between figure skating experts and non-experts was compared to further explore whether neural scaffolding in older adults and inexperienced observers shares a certain degree of similarity (cf., [22]).

Similar action occlusion paradigms have been used previously to examine action observation and prediction in younger adults (e.g., [59]–[63]). Graf et al. [59] and Sparenberg et al. [62], for example, provided evidence that a pure visual encoding and extrapolation of occluded actions do not seem to be sufficient in order to accomplish the task effectively. They showed that prediction performance for temporarily occluded actions that are presented upside-down is considerably impaired compared to the observation of the same actions presented in an upright perspective. The paradigm also proved successful in measuring neural activity in the AON during action prediction [64], [65]. Stadler et al. [65] compared neural activity during the prediction of occluded actions to different action-related control conditions. The authors found that only the dynamic prediction, but not maintenance, of the actions involved activation in parts of the AON. Similarly, Cross et al. [64] showed that activity in the AON is greater during the prediction of partly occluded action sequences compared to the observation of un-occluded segments of the same action sequences.

We hypothesized that the type of observed action sequences modulates activity in the AON. In accordance with previous evidence, we expected to find higher AON activity during the prediction of the movement exercises for which the whole sample was highly experienced with. The less familiar figure skating elements, on the contrary, might be processed in regions beyond the AON due to less precise neural representations in an observer's AON. In addition, we assumed that older adults recruit additional brain regions compared to younger adults, implying less specific internal action representations and/or the reliance on different sources of information in line with the assumptions of STAC [22]. Thus, older adults just as inexperienced observers might perform the task predominantly based on the visual dynamics of the observed actions, which is accompanied with a greater recruitment of visual cortices, respectively. Age-related differences in neural activation patterns might be further modulated by the degree of motor familiarity, for example, in regions known to be involved in episodic memory.

## Materials and Methods

### Ethics statement

The study was approved by the ethics committee of the University of Leipzig and was conducted in accordance with the Declaration of Helsinki. Participants gave written informed consent and received payment for participation.

### Participants

A group of 38 participants, comprising younger and older adults, took part in the fMRI experiment. Three participants (one younger adult and two older adults) were excluded from statistical analyses after medical examination of their anatomical scans in which structural abnormalities were diagnosed that might have an influence on their functional images. In addition, one younger adult was excluded due to experience in professional modern dance for six years in adolescence. The final sample consisted of 19 younger (14 women, mean age = 22.6±2.27 years, range 18–27) and 15 older adults (10 women, mean age = 61.1±5.68 years, range 51–71), *t*(32) = 24.7, *p*<0.001. The majority of the participants already took part in the behavioral action prediction experiment reported in Diersch et al. [8]. One younger adult and four older adults were additionally recruited from the participant database of the MPI for Human Cognitive and Brain Sciences, Leipzig. Before scanning, these participants were invited for a separate testing session in which they completed the relevant questionnaires and performed the behavioral action prediction task to ensure that the whole sample was scanned under the same prerequisites. Time between the two experimental sessions was 5.35 months on average (range 3–8 months). Characteristics of the sample divided by age group are shown in Table 1.

**Table 1:** Characteristics of the sample divided by age group.

	Younger adults	Older adults
	(n = 19)	(n = 15)
Handedness score	92.4 (9.28)	92.5 (9.60)
MMSE score	-	29.2 (0.78)
Years of education	15.7 (3.25)	14.9 (3.11)
DSST raw score	86.0 (14.2)	61.3 (11.0)
DSST standardized score	11.6 (2.77)	10.7 (2.29)
SWT raw score	32.4 (3.24)	32.9 (2.10)
SWT standardized score	0.43 (0.57)	0.47 (0.44)

Values represent mean scores and standard deviations (parenthesized). DSST and SWT values are shown as raw scores and as standardized scores adjusted to the following means: DSST: *M* = 10, *SD* = 3 (age-adjusted); SWT: *M* = 0, *SD* = 1.

All participants were right-handed according to the Edinburgh Handedness Inventory [66] and reported normal or corrected-to-normal vision. None of the participants reported current evidence of any major physical or neurological disease and/or use of medication that might affect blood flow. In addition, participants completed different psychometric tests to ensure that only healthy older adults would be included in the experiment. This allowed an examination of age-related changes in action prediction that are unlikely to be confounded by the effects of any age-associated pathology. None of the older adults showed indications of cognitive impairment as measured by the Mini-Mental State Examination (MMSE; [67]; Maximum score: 30). The groups did not differ with respect to their reported years of education, *t*(32) = 0.68, *p* = 0.500. In addition, fluid intelligence (processing speed) was assessed by means of the Digit Symbol Substitution Test (DSST), a subscale of the Wechsler Adult Intelligence Scale (WAIS-III; [68]). Older adults obtained lower DSST raw scores than younger adults, *t*(32) = 5.53, *p*<0.001, in line with other cognitive aging studies (e.g., [69]). The groups did not differ from each other, when compared with norms appropriate to the participants' age group, *t*(32) = 1.09, *p* = 0.286. Crystallized intelligence (verbal knowledge) was assessed by means of the Spot-the-Word Test (SWT; [70]). In accordance with Park et al. [69], no age-related differences were found, both *t*≤0.46, *p*≥0.648.

With respect to the action sequences used in the experiment, all participants confirmed that they were well able to perform the movement exercises. In addition, 10 of the 34 participants were highly experienced in figure skating (six younger adults and four older adults). The six younger figure skaters (5 women, *M = *21.2, *SD* = 2.23, range 18–24 years) spent on average 11.7 hours per week (*SD* = 7.06) on ice for 14.8 years (*SD* = 2.64). The four older figure skaters (3 women, *M* = 56.0, *SD* = 5.60, range 51–64 years) still performed the sport on a regular basis with 3.50 hours per week (*SD* = 1.29) on ice for 35.0 years (*SD* = 22.4). Two of them pursued a professional career for a period of 14.0 years (*SD* = 6.69) with 14.8 hours per week (*SD* = 7.41) on ice but ended it around the age of 22.7 years (*SD* = 11.0). Characteristics of the sample divided by expertise group can be found in Table S1.

### Stimuli and design

The same video stimuli were used as in the behavioral experiment reported in Diersch et al. [8]. Half of the videos featured classical figure skating elements (e.g., jumps, spins, and step sequences), all of which are listed in the official judging system for single skating specified by the International Skating Union (ISU, www.isu.org). The second set of videos featured simple movement exercises (e.g., running sequences, simple jumps, and spins) that were related to the figure skating sequences as much as possible (e.g., involving rotations) but should be feasible for nearly everyone. Each action was performed by a young male and female athlete (figure skating elements) or non-athlete (movement exercises). The two sets of action sequences were carefully matched with respect to viewing perspective, camera settings, and luminance. The figure skating sequences lasted 11.7 s on average (*SD* = 3.70 s, range 7.40–22.2 s) and the movements exercise sequences 9.00 s on average (*SD* = 0.81 s, range 8.00–10.9 s). In total, 48 different videos consisting of 12 different action sequences from two action categories that were performed by two actors were used in the fMRI experiment.

Each video started with a fixation cross (1000 ms), followed by the beginning of an action sequence. Each action sequence was occluded once for 1000 ms by a grey rectangle at critical time points, for example, when the athlete reached the highest point during the jump. Before each occlusion, the figure skating sequences were visible for 6.24 s (*SD* = 2.54 s, range 3.92–12.4 s) and the movement exercise sequences for 4.50 s (*SD* = 0.76 s, range 3.08–5.92 s) on average, *t*(46) = 3.23, *p* = 0.003. Although the figure skating sequences were visible slightly longer than the movement exercise sequences before they were occluded, there is no reason to assume that this might have influenced the predictive processes in the observers during occlusion. Parkinson et al. [60] recently showed that the prediction of partly occluded actions is nearly unaffected by the length of the action sequences presented before occlusion suggesting that observers engage in prediction very quickly and automatically, even when only a small fraction of human motion is visible. In addition, the critical time frame during which participants were assumed to engage in the internally guided prediction was kept constant across the two action categories (i.e., the duration of occlusion).

**Figure 1:**
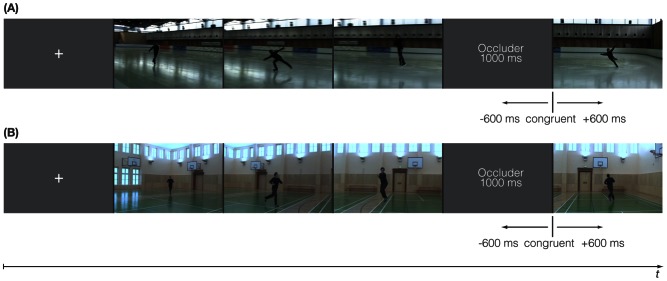
Details of experimental conditions during fMRI scanning. Different action sequences of classical figure skating elements (A) and simple movement exercises (B) were presented. Each video clip started with a fixation cross (1000 ms), followed by the beginning of an action sequence. Then the occluder was presented for 1000 ms, followed by the continuation of the action, that was either congruent or incongruent (±600 ms).

After an occlusion, the action sequences continued immediately. The continuations after occlusion were either congruent or incongruent (i.e., 600 ms too early or too late, see Figure 1 for an example from each action category). Based on the results of the previous behavioral study, in which incongruent continuations of ±400 ms and ±800 ms were used and the prediction sensitivity of the different groups (i.e., response slopes) was analyzed, a temporal shift of ±600 ms was chosen to examine age-related differences in the neural representation of the different action sequences at an intermediate level of difficulty (cf., [8]).

The action sequences were presented in full color with a resolution of 1024×768 pixels and a frame rate of 25 frames per second using a back projection system in which a LCD projection on a screen in the back of the scanner was reflected by a mirror placed above the participants' eyes. The software “Presentation” (Neurobehavioral Systems, Albany, CA) was used to control stimulus presentation and behavioral data collection.

### Task and procedure

For MRI scanning, participants were provided with ear-plugs and headphones to reduce scanner noise. Vision was corrected with MRI-compatible plastic goggles, if necessary. The participants' task was to judge for each observed action sequence whether the continuation after occlusion was correct or not by pressing on one of two response keys (left key: correct, right key: incorrect) with their index and middle finger on a response device that was placed in their right hand. Participants were instructed to respond as quickly and accurately as possible as soon as the action sequence continued after occlusion. An event-related design was used to measure neural responses during the prediction of observed action sequences.

Prior to the functional run, participants completed a short familiarization and training phase in the scanner during the acquisition of initial control sequences. This allowed them to accommodate to the task and the scanner environment. The familiarization started with two action sequences from each action category that were presented without occlusion and two action sequences from each action category that were presented with occlusion. These action sequences (four different action sequences from each action category) were also presented in a subsequent training phase, in which participants were required to perform the prediction task and received feedback of their performance. The training phase consisted of 16 trials per action category (32 in total). The remaining eight action sequences from each action category that were used in the actual test phase were presented once without occlusion before the functional run started.

The functional run, in which no feedback was given, consisted of 80 trials (8 action sequences×2 actors×5 repetitions) per action category (160 in total), in which the congruent and incongruent continuations were presented 40 times each. The action sequences were presented in blocks consisting of eight videos from one action category, in which no action was repeated after one another, resulting in 10 blocks from each category (20 in total). The continuations after occlusion were randomized separately with the restriction that the same continuation should not be presented more than two times in a row with a maximum of three congruent or incongruent continuations after one another. Within each action category, the congruent continuation was presented twice as often as the two incongruent continuations (i.e., too early and too late), which resulted in an equal number of congruent and incongruent continuations. The order of the videos and continuations was counterbalanced across participants. After each video block, a resting baseline showing a black screen with a grey fixation cross was presented for 8–12 s, pseudo-logarithmically distributed. The functional run lasted approximately 32 min.

Scanning was performed on a 3T TIM Trio scanner (Siemens, Erlangen, Germany) with a 12-channel head array coil. Functional images were acquired with a gradient echo echo-planar imaging (EPI) sequence with TR = 2000 ms, TE = 30 ms, flip angle = 90°, and acquisition bandwidth = 1815 Hz/pixel. The matrix acquired was 64×64 voxels with a FoV of 192 mm×192 mm, resulting in an in-plane resolution of 3 mm×3 mm. Twenty-six axial slices allowing for full-brain coverage were acquired in ascending order with slice thickness = 4 mm and interslice gap = 1 mm. Slices were oriented parallel to the bicommisural plane (AC-PC). A set of 1020 functional images was collected in a single functional run. In addition to functional imaging, high-resolution anatomical images were acquired using a T1-weighted 3D magnetization-prepared rapid gradient echo (MPRAGE) sequence with selective water excitation and linear phase encoding [71]. Anatomical scanning was performed using a sagittal slice orientation with the following imaging parameters: TI = 650 ms, TR = 1300 ms, TE = 3.5 ms, flip angle = 10°, acquisition bandwidth = 190 Hz/pixel, image matrix = 256×240 voxels, FoV = 240 mm×256 mm, spatial resolution = 1 mm×1 mm×1 mm, 2 acquisitions. All MR datasets obtained in the present study are stored in anonymized form in a database of the MPI for Human Cognitive and Brain Sciences, Leipzig.

### Data analysis

Data analysis was performed using the SPM8 software package (Wellcome Department of Imaging Neuroscience, London, UK) with Matlab 7 (Mathworks, Natick, MA). Preprocessing of the EPI volumes included correction for motion and distortion, slice timing, as well as normalization to the standard MNI space using the unified segmentation approach [72]. Finally, spatial smoothing was done using an 8 mm full-width at half maximum isotropic Gaussian kernel. A two-level random effects approach as implemented in SPM8 was used for the statistical analyses. On the individual level, observed action category as a function of continuation after occlusion was modeled for each participant as separate events convolved with the standard hemodynamic response function: figure skating sequences that continued congruently, figure skating sequences that continued incongruently, movement exercise sequences that continued congruently, and movement exercise sequences that continued incongruently. This resulted in an equal number of relevant events in each condition.

The respective beginning of occlusion was defined as the target event in order to capture the time in which participants were assumed to engage in the internally guided prediction of the occluded action sequences. The results of our previous study suggested that the sensitivity in action prediction is lower in older adults compared to younger adults and in non-experts compared to experts as evidenced by a larger temporal range during which the continuations after occlusion were predominantly perceived as being just-in-time (i.e., resulting in higher error rates; [8]). In the present study, we aimed to examine the neural effects of aging and sensorimotor experience during action prediction that might accompany these differences in behavioral efficiency. Thus, both correct and incorrect trials from each critical condition were incorporated in the fMRI analyses, which also ensured that the same number of events would be included in the analyses of the between-subject effects. In addition, given that neuroimaging data and behavioral data may both be considered as effects of an underlying functional difference (i.e., aging), excluding incorrect responses from the fMRI analysis might also remove age-related differences at the neural level (cf., [73]). The time of the button press was modeled as additional event to control for the effects of finger movements. Each baseline condition was modeled as a boxcar with the respective duration. Confounding factors from head movement, that is, six rotational and translational parameters from the rigid body transformation, obtained during image realignment, were included in the model as covariates of no interest. A high-pass filter at 1/100 Hz was used to remove low-frequency fluctuations of the MR signal. Whole brain analyses were conducted using a voxel-wise threshold of *p*<0.001 and a minimum cluster size of 10 voxels. To control for false positive results, analyses focus on brain regions reaching a cluster-corrected significance threshold of *p*<0.05 (FWE corrected).

On the first level, the effects of each action category, collapsed across the continuations after occlusion, were compared to baseline and directly to each other, as were the interactions with continuation after occlusion by computing contrast images combining the parameter estimates of the corresponding experimental conditions. On the second level, those contrast images were fed into one-sample *t*-tests to perform inference statistics across the whole sample. Between-subject effects were tested using the general linear model as implemented in SPM. Due to the small sample size of figure skating experts in the two age-groups, two-sample *t*-tests instead of a full factorial design were used to examine the effects of age while correcting for non-sphericity through assuming measurement independence und unequal variance between groups. More specifically, differences between older and younger adults on the respective first-level comparisons were examined while taking experience in figure skating as a covariate of no interest into account. In addition, differences between figure skating experts and non-experts were tested accordingly while including age group as covariate of no interest. Although this did not allow for a direct investigation of interactions between age and experience in figure skating, the effects of motor familiarity were examined as a function of observed action category given that all of the participants were highly experienced with respect to the observed movement exercises. Significant group differences were further examined separately within the respective groups by means of one-sample *t*-tests of the individual contrast images. Anatomical localization of all activations was aided by the Anatomy Toolbox in SPM8 [74] in combination with the Atlas of the Human Brain [75].

## Results

### Behavioral results

Prediction performance was calculated as proportion of correct responses of every group on congruent and incongruent continuations for each action category with an equal number of trials for each condition. The proportion of correct responses was submitted into an ANOVA with action category (figure skating elements, movement exercises) and continuation after occlusion (congruent, incongruent) as repeated measures variables and age group (younger adults, older adults) and expertise group (figure skating experts, non-experts) as between-subject variables. The ANOVA revealed a significant main effect of continuation after occlusion, *F*(1,30) = 11.1, *p* = 0.002, η_p_^2^ = 0.271, and a significant main effect of age group, *F*(1,30) = 18.8, *p*<0.001, η_p_^2^ = 0.385. This was modulated by a significant interaction between continuation after occlusion and age group, *F*(1,30) = 4.99, *p* = 0.033, η_p_^2^ = 0.143. The performance of the groups did not differ significantly when the actions continued congruently (younger adults: *M* = 67.6%, *SD* = 13.0%; older adults: *M* = 61.7%, *SD* = 17.6%), *t*(32) = 1.12, *p* = 0.271. On incongruent continuations, however, the performance of older adults (*M* = 43.0%, *SD* = 11.1%) was significantly lower than the performance of younger adults (*M* = 62.4%, *SD* = 9.96%), *t*(32) = 5.36, *p*<0.001. Prediction performance of the two age groups did differ significantly from chance level for both types of continuation after occlusion, all *t*≥2.45, *p*≤.028. In addition, a significant main effect of expertise group was found, *F*(1,30) = 4.90, *p* = 0.035, η_p_^2^ = 0.140, which was modulated by a significant interaction between action category, age group, and expertise group, *F*(1,30) = 8.04, *p* = 0.008, η_p_^2^ = 0.211, as well as a significant interaction between action category, continuation after occlusion, age group, and expertise group, *F*(1,30) = 4.26, *p* = 0.048, η_p_^2^ = 0.124. This implies that the performance of the groups differed as a function of observed action category and continuation after occlusion.

To further examine these interactions, follow-up ANOVAs with continuation after occlusion (congruent, incongruent) as repeated measures variable and age group (younger adults, older adults) and expertise group (figure skating experts, non-experts) as between-subject variables were conducted for each action category separately. For the figure skating elements, a significant main effect of continuation after occlusion, *F*(1,30) = 8.32, *p* = 0.007, η_p_^2^ = 0.217, confirmed that performance was better for congruent (*M* = 65.0%, *SD* = 18.3%) than incongruent continuations (*M* = 53.3%, *SD* = 16.3%). In addition, a significant main effect of age group, *F*(1,30) = 18.5, *p*<0.001, η_p_^2^ = 0.382, and a main effect of expertise group, *F*(1,30) = 4.54, *p* = 0.041, η_p_^2^ = 0.132, was found. Thus, not only young age (younger adults: *M* = 65.3%, *SD* = 12.3%; older adults: *M* = 51.4%, *SD* = 7.70%) but also experience in figure skating (experts: *M* = 66.0%, *SD* = 16.9%; non-experts: *M* = 56.3%, *SD* = 9.21%) had a positive effect on prediction accuracy during the observation of figure skating elements.

The follow-up ANOVA for the movement exercises also showed a significant main effect of continuation after occlusion, *F*(1,30) = 11.2, *p* = 0.002, η_p_^2^ = 0.272, and a significant main effect of age group, *F*(1,30) = 11.1, *p* = 0.002, η_p_^2^ = 0.270. As in the overall analysis, this was modulated by a significant interaction between continuation after occlusion and age group, *F*(1,30) = 6.17, *p* = 0.019, η_p_^2^ = 0.170. Older (*M* = 63.6%, *SD* = 18.3%) and younger adults (*M* = 66.0%, *SD* = 11.0%) did not differ significantly in their performance on congruent continuations, *t*(32) = .46, *p* = 0.648. On incongruent continuations, older adults' performance dropped significantly (*M* = 42.9%, *SD* = 12.8%) compared to younger adults (*M* = 63.3%, *SD* = 8.80%), *t*(32) = 5.51, *p*<0.001.

The results show that older adults predicted the observed action sequences less precisely compared to younger adults, even when they were familiar with the observed actions. They incorrectly perceived incongruent continuations predominantly as still being congruent, which is in line with the results of our previous study suggesting that the temporal sensitivity in action prediction declines with age (cf., [8]). Moreover, sensorimotor experience in figure skating exerted a positive influence on the performance of experts compared to non-experts of the same age group during the observation of the figure skating elements. Together, the behavioral data suggest that all groups attended to the action sequences and engaged in action prediction in the manner that was hypothesized during fMRI scanning.

### Neuroimaging results

#### Effects of predicted action category

**Figure 2:**
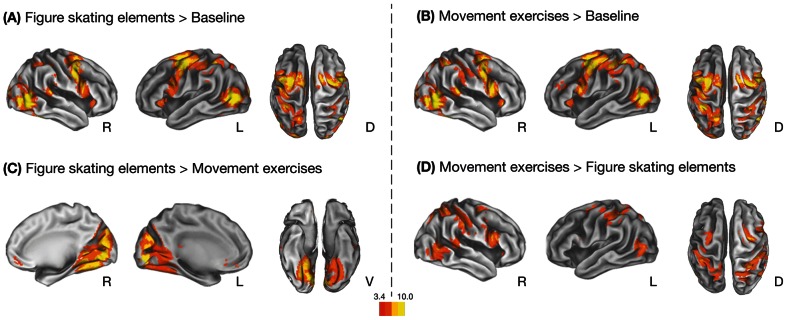
Main effects of observed action category collapsed across the whole sample. Brain regions that showed greater activation during the prediction of figure skating elements (A) and movement exercises (B) compared to baseline, and direct comparisons between the action categories (C and D). Results were calculated using a voxel-wise threshold of *p*<0.001 and a minimum cluster size of 10 voxels. Abbreviations: R – Right Hemisphere; L – Left hemisphere; D – Dorsal; V – Ventral.

The prediction of both types of action sequences compared to baseline resulted in bilateral activity in frontal, parietal, occipitotemporal, and occipital regions as well as in some subcortical structures (Fig. 2A,B and Table S2). The direct comparison between the action categories revealed remarkable differences. Compared to movement exercises, the visual cortex and the medial orbitofrontal cortex (OFC) were more engaged during the prediction of figure skating elements (Fig. 2C and Table 2A). In contrast, the premotor, parietal and occipitotemporal regions of the AON were preferentially activated during the prediction of movement exercises compared to figure skating elements (Fig. 2D and Table 2B). For the movement exercises only, the right posterior superior temporal sulcus (pSTS) differentiated between incongruent and congruent continuations after occlusion (Table 2C). Thus, different regions of the AON showed selectivity for the generally familiar movement exercises whereas visual and frontal areas responded stronger to the less familiar figure skating elements.

**Table 2:** Effects of predicted action category and continuation after occlusion.

				MNI coordinates (mm)			*t* value	*p* value
Anatomical region	Putative functional name	BA	Cluster size	*x*	*y*	*z*	*df = *[1,33]	(corr.)
**(A) Figure skating elements > Movement exercises**								
Midline Calcerine Gyrus	V1	17	3615	0	−88	−5	11.84	< 0.001
R Lingual Gyrus	V2/V3	18		12	−73	−5	11.38	
R Fusiform Gyrus		19		27	−64	−11	9.05	
R Superior Frontal Gyrus	vmPFC	10	117	12	50	4	4.78	0.022
R Medial Orbitofrontal Gyrus	OFC	32		12	41	−8	4.33	
L Medial Orbitofrontal Gyrus	OFC	32		−3	41	−8	3.96	
**(B) Movement exercises > Figure skating elements**								
R Intraparietal Sulcus	IPS	7/40	1791	36	−49	55	8.09	<0.001
R Superior Temporal Gyrus	pSTS	22		54	−40	10	7.38	
R Supramarginal Gyrus	IPL	40		60	−22	43	7.26	
R Precentral Gyrus	PMd	6	761	30	−10	52	8.01	<0.001
R Inferior Frontal Gyrus (pars opercularis)	PMv	44		57	11	25	7.53	
R Middle Frontal Gyrus	PMd	6		24	8	43	4.81	
L Inferior Parietal Lobule	IPL	7/40	1133	−39	−37	46	7.12	<0.001
L Middle Frontal Gyrus	PMd	6		−27	−10	55	6.73	
L Superior Parietal Lobule	SPL	7		−18	−64	58	6.16	
L Middle Occipital Gyrus	V5/hMT+	39	194	−48	−73	4	6.78	0.003
**(C) Movement exercises: incongruent > congruent**								
R Middle Temporal Gyrus	pSTS	22	151	60	−49	10	4.90	0.022
R Middle Temporal Gyrus	pSTS	22		54	−43	7	4.62	

Regions activated during the prediction of figure skating elements compared to movement exercises (A) and vice versa (B). Regions activated during incongruent compared to congruent continuations after occlusion during the prediction of movement exercises are shown in section (C). Results are collapsed across the whole sample using a voxel-wise threshold of *p*<0.001 and a minimum cluster size of 10 voxels. Only clusters are reported that reached cluster-corrected significance of *p*<0.05, FWE corrected. Up to three local maxima are listed when a cluster has multiple peaks more than 8 mm apart. Abbreviations for brain regions: V1, visual area V1/striate visual cortex; V2, visual area V2/prestriate visual cortex; V3, visual area V3/extrastriate visual cortex; vmPFC, ventromedial prefrontal cortex; OFC, orbitofrontal cortex; IPS, intraparietal sulcus; pSTS, posterior superior temporal sulcus; IPL, inferior parietal lobule; PMd, dorsal premotor cortex; PMv, ventral premotor cortex; SPL, superior parietal lobule; V5/hMT+, visual area V5/extrastriate visual cortex/middle temporal.

#### Effects of age group

**Figure 3:**
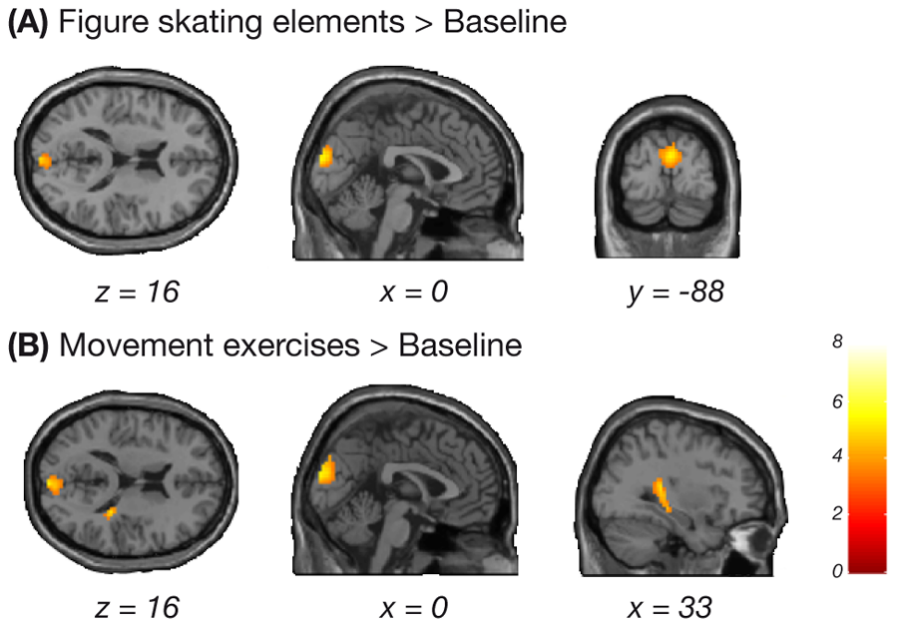
Brain regions more activated in older adults compared to younger adults. Patterns of brain activation during the prediction of figure skating elements (A) and movement exercises (B) compared to baseline. Results were calculated using a voxel-wise threshold of *p*<0.001 and a minimum cluster size of 10 voxels. Only clusters are shown that reached cluster-corrected significance of *p*<0.05, FWE corrected.

In order to evaluate regions in which activation varied as a function of age group, older and younger adults were compared to each other while the factor experience in figure skating was included as covariate of no interest. Compared to younger adults, older adults showed a greater recruitment of the prestriate and extrastriate visual cortex, bilaterally centered in the cuneus, for the figure skating elements as well as the movement exercises compared to the baseline condition (Fig. 3A,B and Table 3A,B). During the prediction of movement exercises, older adults engaged an additional region in the right posterior hippocampus extending to the right caudate more than younger adults (Fig. 3B and Table 3B). The reverse contrasts did not reveal any significant clusters that were more activated in younger adults compared to older adults. This confirms that older adults recruited areas beyond the AON, which younger adults did not, during action prediction.

**Table 3:** Main effects of age group for each predicted action category compared to baseline.

				MNI coordinates (mm)			*t* value	*p* value
Anatomical region	Putative functional name	BA	Cluster size	*x*	*y*	*z*	*df = *[1,31]	(corr.)
**(A) Figure skating elements > Baseline**								
R Cuneus	V2/V3	18	117	3	−91	22	5.28	0.030
R Cuneus	V3	19		3	−85	37	3.52	
**(B) Movement exercises > Baseline**								
R Cuneus	V2/V3	18	160	3	−91	22	5.76	0.009
R Insula		13	107	33	−34	13	4.85	0.039
R Hippocampus				36	−28	−8	4.67	
R Caudate				21	−25	22	4.17	

Regions more activated in older adults compared to younger adults while controlling for expertise group during the prediction of figure skating elements (A) and movement exercises (B) compared to baseline. Results were calculated using a voxel-wise threshold of *p*<0.001 and a minimum cluster size of 10 voxels. Only clusters are reported that reached cluster-corrected significance of *p*<0.05, FWE corrected. Up to three local maxima are listed when a cluster has multiple peaks more than 8 mm apart. Abbreviations for brain regions: V2, visual area V2/prestriate visual cortex; V3, visual area V3/extrastriate visual cortex.

**Figure 4:**
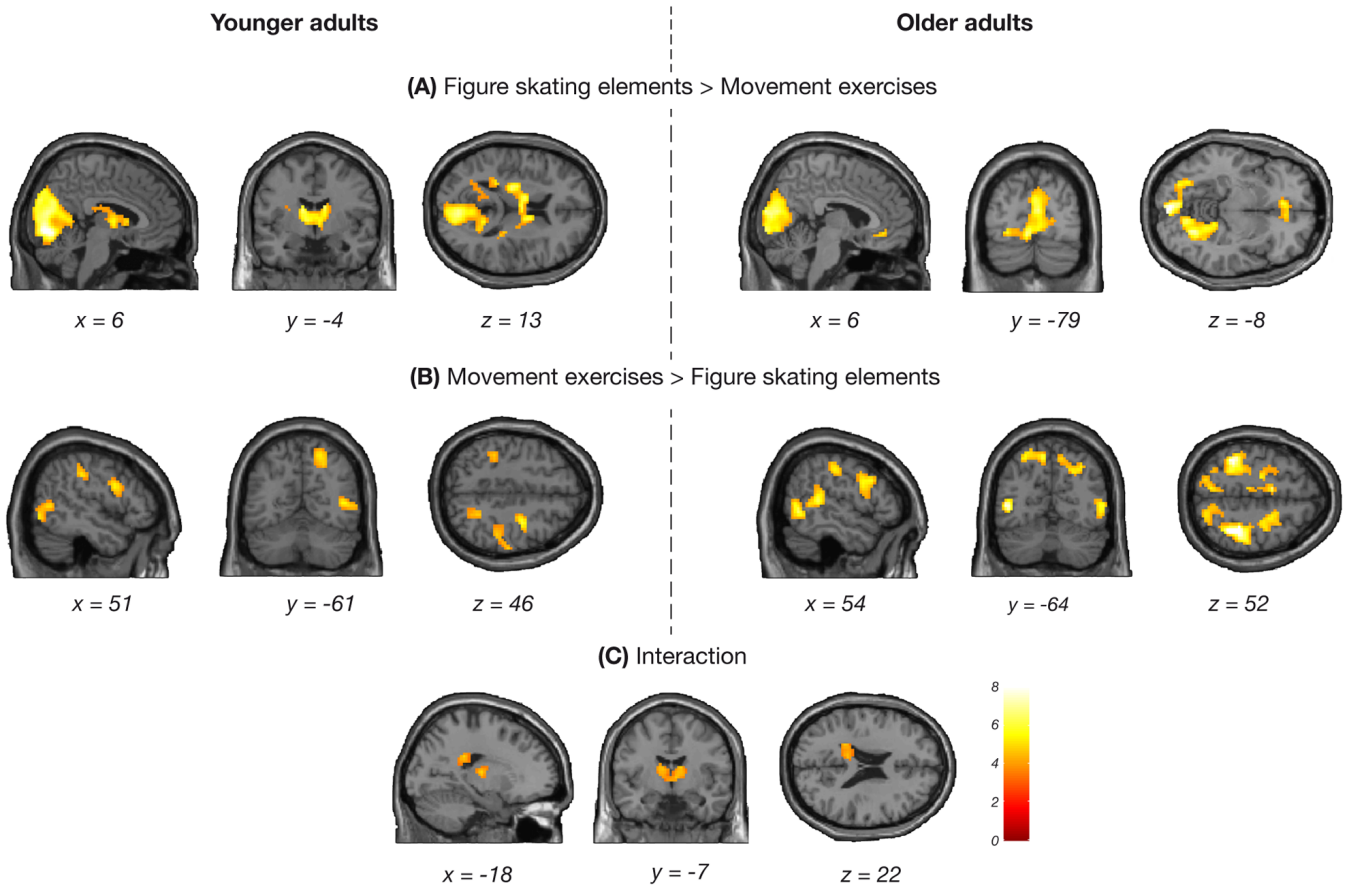
Interaction between predicted action category and age group. Brain regions that showed greater activation in younger (left panels) and older adults (right panels) during the prediction of figure skating elements compared to movement exercises (A) and vice versa (B). The interaction is shown in panel (C). Results were calculated using a voxel-wise threshold of *p*<0.001 and a minimum cluster size of 10 voxels. Only clusters are shown that reached cluster-corrected significance of *p*<0.05, FWE corrected.

In addition, a significant interaction between predicted action category and age group was found in the left caudate and the bilateral thalamus together with the left posterior cingulate cortex (PCC; Fig. 4C and Table 4A). To examine this interaction further, a comparison between both types of action sequences was conducted within each age group. The results are illustrated in the upper panels of Figure 4 and a complete listing of suprathreshold activations in each age group can be found in Table S3. This analysis revealed that the interaction was due to the younger adults, who showed a greater recruitment of the visual cortex that extended to the PCC and the thalamostriatal network during the prediction of the figure skating elements compared to movement exercises. In older adults, the same comparison revealed only the visual cortex and the medial OFC. In contrast, premotor, parietal and occipitotemporal regions of the AON that were largely confined to the right hemisphere were more activated in younger adults when they predicted movement exercises compared to figure skating elements. In older adults, similar regions were found for the same contrast, although mainly bilaterally distributed. No age-related activation differences that reached cluster-corrected significance were found for the interactions between action category and continuation after occlusion.

**Table 4:** Interactions between predicted action category and group.

				MNI coordinates (mm)			*t* value	*p* value
Anatomical region	Putative functional name	BA	Cluster size	*x*	*y*	*z*	*df = *[1,31]	(corr.)
**(A) Interaction with age group**								
L Thalamus			271	−21	−16	10	5.36	< 0.001
Midline Thalamus				0	−22	4	5.15	
L Caudate				−9	−4	13	4.60	
L Posterior Cingulate Gyrus	PCC	23/31	105	−18	−37	28	4.70	0.027
L Posterior Cingulate Gyrus	PCC	23		−24	−43	19	4.57	
L Posterior Cingulate Gyrus	PCC	23		−9	−37	22	4.42	
**(B) Interaction with expertise group**								
L Caudate			195	−15	−22	13	4.58	0.002
L Caudate				−18	−7	16	4.37	
L Thalamus				−9	−10	7	4.31	

Interactions between action category and age group (A) and expertise group (B). Results were calculated using a voxel-wise threshold of *p*<0.001 and a minimum cluster size of 10 voxels. Only clusters are reported that reached cluster-corrected significance of *p*<0.05, FWE corrected. Up to three local maxima are listed when a cluster has multiple peaks more than 8 mm apart. Abbreviations for brain regions: PCC, posterior cingulate cortex.

### Effects of expertise group

**Figure 5:**
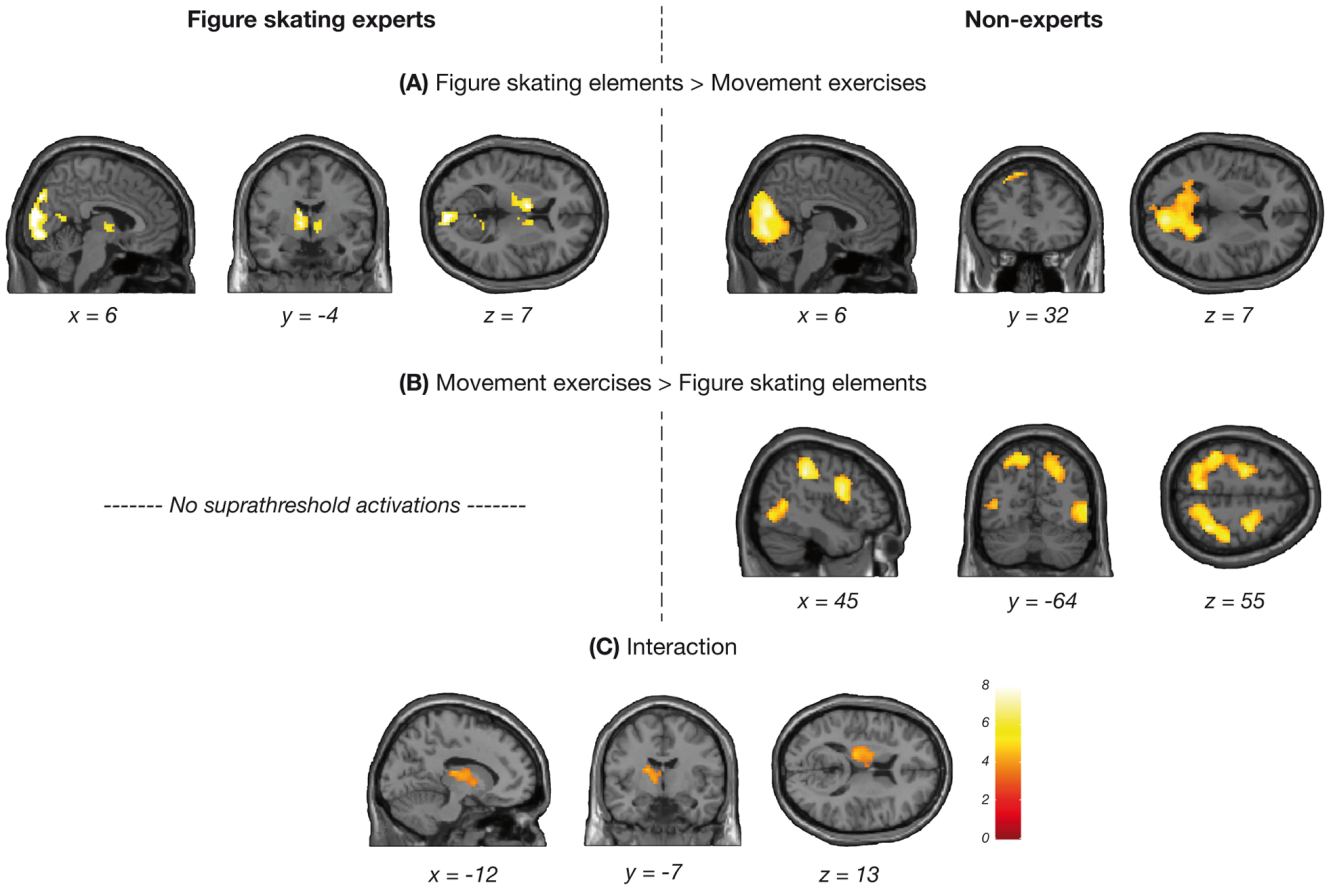
Interaction between predicted action category and expertise group. Brain regions that showed greater activation in figure skating experts (left panels) and non-experts (right panels) during the prediction of figure skating elements compared to movement exercises (A) and vice versa (B). The interaction is shown in panel (C). Results were calculated using a voxel-wise threshold of *p*<0.001 and a minimum cluster size of 10 voxels. Only clusters are shown that reached cluster-corrected significance of *p*<0.05, FWE corrected.

In order to explore differences in neural activation patterns as a function of experience in figure skating, figure skating experts and non-experts were compared to each other while the factor age group was included as covariate of no interest. Experts and non-experts did not differ significantly from each other for both types of action sequences compared to baseline. Interestingly, a significant interaction between predicted action category and expertise group was found again in the left caudate and the left thalamus (Fig. 5C and Table 4B).

The results of the comparisons between the types of action sequences within each expertise group are depicted in the upper panels of Figure 5. A complete listing of suprathreshold activations in each expertise group is provided in Table S4. This analysis revealed that the interaction was due to the figure skating experts, who engaged the bilateral caudate and thalamus together with the early visual cortex more during the prediction of figure skating elements compared to movement exercises. The non-experts, in contrast, showed a greater recruitment of the whole visual cortex and the superior frontal gyrus for this comparison. The reverse contrast did not reveal any suprathreshold activations in experts whereas non-experts engaged premotor, parietal and occipitotemporal regions of the AON during the prediction of movement exercises compared to figure skating elements. No expertise-related activation differences that reached cluster-corrected significance were found for the interactions between action category and continuation after occlusion.

## Discussion

The present study aimed to identify age-related differences in neural activation patterns during the prediction of action sequences that varied in their degree of motor familiarity (i.e., classical figure skating elements and simple movement exercises). Further, the possible role and consequences of neural scaffolding in older adults during action prediction was explored through the inclusion of observers who differed in their degree of sensorimotor experience with the observed actions. In addition, we aimed to clarify the link between AON activity and motor familiarity during action prediction in general. With respect to the AON, our results show that activity in different regions of this network was modulated by sensorimotor experience with the observed actions. Whereas the sensorimotor and occipitotemporal cortices that compose the AON showed more activation for the generally more familiar movement exercises, the prediction of figure skating elements resulted in increased engagement of the visual cortex and the medial OFC. Compared to younger adults, older adults recruited visual regions while performing the prediction task. Older adults also showed greater recruitment of the hippocampus and caudate when predicting actions that were familiar to them. During prediction of the figure skating elements, the caudate together with the thalamus seemed to play an important role in younger observers. In addition, our data indicate that this might have been similarly the case in observers who possessed sensorimotor experience in figure skating. However, due to the small sample size of the figure skating experts in particular, the interpretation of these findings has to be taken with caution. Each of these results and their implications will be considered in turn.

### Modulation of AON activity as a function of predicted action category

The prediction of both types of action sequences was accompanied by robust AON activation compared to baseline, in line with many others studies showing that this network is involved in the anticipation of observed actions (e.g., [32]–[35], [64], [65], [76]).

The direct comparison between the different action sequences revealed that AON activation was increased for the movement exercises, which is in accordance with other studies that found enhanced activity in these regions for familiar actions compared to actions that are not in motor repertoire of the observer (e.g., [47], [48], [50]–[53]). However, the precise relation between level of familiarity and level of activation in the AON is still a matter of debate because some studies also demonstrated decreased AON activity for familiar compared to unfamiliar actions (e.g., [77]–[80]). To reconcile such seemingly discrepant findings, Cross et al. [80] have proposed a nonlinear relationship between motor familiarity in the observer and AON activity that follows a u-shaped function. According to this model, highly unfamiliar actions produce a greater prediction error than actions of intermediate familiarity. This greater prediction error results in increased AON activity due to increased processing demands between the different regions of the AON. In contrast, highly familiar actions might also lead to enhanced AON activity compared to actions of intermediate familiarity, but for a different reason. Here, participants have generated extremely exact predictions due to a high degree of motor expertise. Any small deviations from such precise predictions might amplify the response within the AON if the sensory input does not exactly match the predicted consequences.

**Figure 6:**
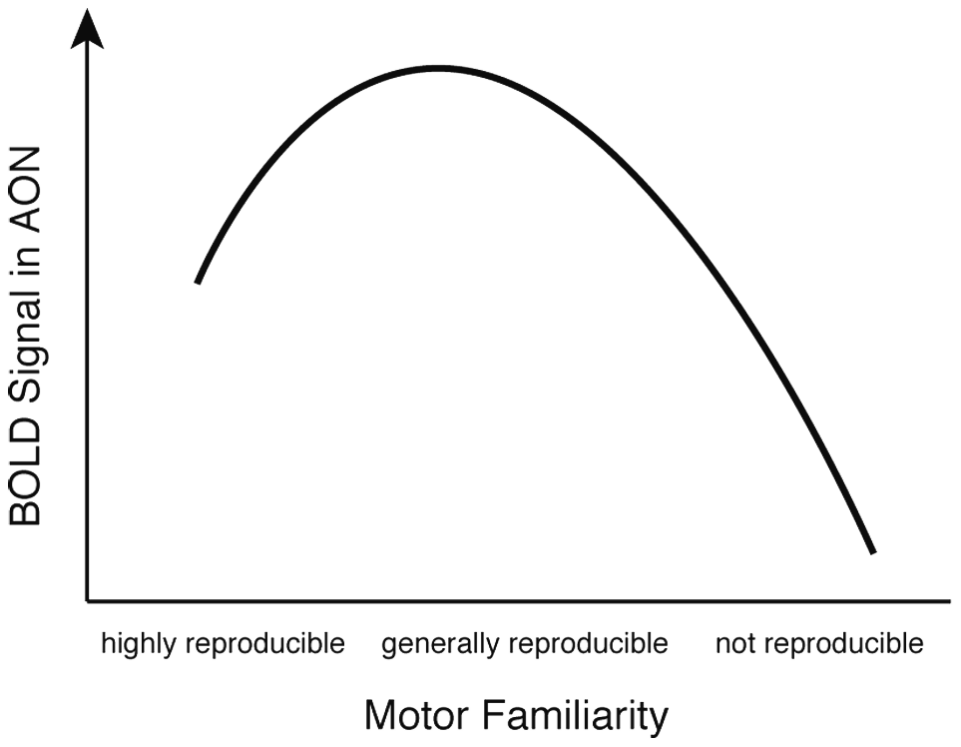
Adapted model of the hypothesized relationship between motor familiarity and activity in the AON.

According to this proposal, here we might have expected increased AON activity for the less familiar figure skating elements in comparison to the generally familiar movement exercises. These discrepancies in the present as well as across previous studies might be related to the respective definition of unfamiliar actions. For example, unfamiliar actions might be defined as those that are not regularly seen, but are generally executable by the observer, such as unusual hand gestures. They might be also defined as not in the motor repertoire of the observer at all, such as figure skating elements for observers who have never ice skated before. In addition, for movement exercises, only one region of the AON, the pSTS, showed stronger activity when observing incongruent compared to congruent continuations after occlusion. Such a finding is in line with evidence that the STS is involved in the perception of biological motion and contains cells with predictive properties that are sensitive to movements that deviate from expectations [81], [82]. One should also note that this activation was close to a occipitotemporal region previously identified as the extrastriate body area (EBA; [83]). The EBA has been implicated in the evaluation of biomechanical constraints in visual body processing in action-related contexts (e.g., [64], [84]). In addition, higher activity in this region was found in experts compared to non-experts during action prediction [54]. This suggests that regions within but also beyond the AON might be involved in action prediction depending on the characteristics of the observed actions and the observers' level of motor familiarity. We therefore propose an adaptation to the model put forth by Cross et al. [80] that also considers unfamiliar actions that the observer cannot reproduce without extensive training in comparison to unfamiliar actions for which the observed kinematics might be inferable and at least to some extent reproducible (see Fig. 6).

Within this adapted model, a match between observed and predicted representation of highly familiar actions should be associated with a small prediction error and, thus, intermediate AON activity. Less familiar actions might result in higher AON activity due to a less precise prediction of them that needs to be constantly updated based on the actual sensory input to minimize a larger prediction error. The use of these predictive representations might become less efficient the less familiar an observed action is, until an internal action representation will not be present at all. In these cases, the observed actions might be represented in a rather multimodal way that is supported by the recruitment of regions beyond the classical AON (cf., [52], [53], [55], [56]).

In line with this, our data show that the less familiar figure skating elements were processed more in the visual cortex and in the medial OFC, a region that is known to be involved in adaptive decision making in unpredictable situations [85], [86]. Although special care was taken to match both types of action sequences as much as possible in terms of involved body kinematics, the figure skating elements were executed at a considerably faster speed than the movement exercises. This might have skewed the perceptual complexity and in turn biased the demands on the predictive processes in the observer to some extent. However, the activation in the medial OFC implies that differences in low-level visual features might only partially explain the observed differences in neural activity. This assumption is further supported by the observed group differences in the present study that are discussed below. In addition, given that the action sequences in the present study fulfilled a rather dichotomous criterion with respect to motor familiarity (i.e., either generally familiar or highly unfamiliar actions), further research is clearly warranted that tests the implications of the proposed model more directly. For example, including actions from a continuous range of familiarity would be useful in order to gain a better understanding of how individual differences in motor (and/or visual) familiarity may modulate predictive processing during action observation (see also [87] for another variation of familiarity). Although beyond the scope of this study, the precise level of AON involvement most likely depends on the specific task demands as well. For instance, whether an action is just passively observed or whether the observer intents to infer its meaning or predicts its time course poses important differences in the neural processing demands in the observer (cf., [64], [65], [88]).

### Action prediction in the aging brain

With respect to the AON in particular, we did not find age-related activation differences that reached cluster-corrected significance as in Nedelko et al. [58]. However, the comparison between the action categories within each age group indicated that AON activity was mainly right lateralized in younger adults and bilaterally active in older adults during the prediction of movement exercises compared to figure skating elements. This pattern of activity change has been observed frequently in different cognitive domains and is referred to as hemispheric asymmetry reduction in old adults (HAROLD), suggesting that cortical recruitment under similar conditions tends to be less lateralized in older adults [19].

The results further show that older adults recruited regions beyond the AON, which younger adults did not, while performing the task. No matter what type of action sequence was observed, when comparing to the baseline condition, older adults showed greater recruitment of prestriate and extrastriate visual cortex compared to younger adults. In younger adults, these early visual areas, such as V2, have been shown to be recruited in mental imagery tasks that require the anticipation of objects or scenes that one is about to perceive [89]. In older adults, however, evidence suggests that the neural representation of sensory input becomes less distinct with age, arguing for an age-related neural dedifferentiation in relevant areas [25], [29]. In line with this assumption, behavioral evidence suggests that the correlation between sensory and cognitive abilities increases with age [90]. The increased activation in the visual cortex might therefore partly reflect less specific sensory representations of the observed actions among older adults. These less distinct sensory representations might have been matched with motor representations that also get less selective with age (cf., [24], [26], [28], [30], [31]).

However, when examined separately, the non-experts in figure skating also demonstrated greater recruitment of a large cluster in the visual cortex during prediction of figure skating elements compared to movement exercises. In addition, Olsson et al. [52] and Wright et al. [53] found higher activation in very similar visual areas in non-experts compared to experts during action imagery and observation. This implies that the engagement of visual regions in older adults cannot be solely explained by neural dedifferentiation in the aging mind. The findings rather suggest that the brain's response to challenges that are due to aging or the exposure to unfamiliar material may indeed be similar during the prediction of others' actions (cf., [22]).

Older adults showed additional activation within the medial OFC during the prediction of the figure skating elements compared to movement exercises. The OFC has been shown to play an important role in the top-down modulation of visual processing through the generation of initial predictions about likely interpretations of the visual input in younger adults [91]. In their fMRI study on expert-novice differences during the prediction of basketball throws, Abreu et al. [54] recently found that orbito-frontal regions are specifically linked to correct action prediction in observers who are not familiar with the shown actions. Thus, one might speculate that also the older adults of the present study relied more on these higher-order regions that are involved in adaptive decision-making during the prediction of actions that were less familiar to them.

For the movement exercises, compared to younger adults, older adults recruited an additional cluster in the right hippocampus extending to the caudate compared to baseline. Neurobiological evidence suggests that these regions form a functional network that is involved in flexible decision-making with the hippocampus generating predictive (spatial) representations and the caudate learning and anticipating action-outcome contingencies (e.g., [92], [93]). In addition, hippocampal activation has been found during episodic imagination of the future that is based on a recombination of past episodic events [94], [95]. Recent evidence suggests that this functional differentiation, with the hippocampus mediating explicit/declarative memory and the striatum mediating implicit/procedural memory, decreases with advancing age [96]. Thus, if older adults in the present study were familiar with the observed actions, they seemed to use learned action-outcome contingencies as well as multimodal representations of these actions stored in episodic memory to evaluate the sensory input. Age-related declines in neural selectivity in these regions together with a less efficient use of the own sensorimotor system might in turn have resulted in difficulties to recreate the observed action sequences in necessary detail in order to predict their exact time-course. This is further supported by findings that the reconstruction of episodic details comprising past and future events is reduced in older adults, which is linked to activity changes in medial temporal regions [97], [98].

Taken together, the data provide evidence for age-related neural scaffolding in relevant areas during action prediction that is modulated by the degree of motor familiarity with the observed actions. Older adults may have relied predominantly on the visual dynamics of the observed actions during the occlusion period instead of effectively exploiting the sensorimotor matching properties of the AON. Even though it was beyond the scope of this study to examine the neural correlates of successful action prediction within the single groups, it may provide important insights on how the process of action prediction is generally implemented in the aging brain. In the present study, neural activity was measured at the beginning of occlusion to capture the time in which participants were assumed to internally predict the occluded action sequences. Their explicit decision about the continuation after occlusion, however, occurred several seconds later. Future research is therefore needed in order to specify how age-related changes at the neural level are associated with declines in behavioral performance and how predictive coding may actually change with advancing age. In the context of predictive coding, it is argued that predictive processing during perception takes place at multiple levels in the cortical hierarchy (cf., [39], [99], [100]). For example, dopamine has recently been implicated in modulating the precision of prediction errors (or uncertainty) at different levels in the sensorimotor hierarchy [101]. Changes in neurotransmitter systems such as the dopaminergic system have been linked to neural dedifferentiation in older adults [21]. Thus, one might speculate that the age-related loss in selectivity in sensory representations and/or prior expectations reflects changes in neurotransmitter function in the aging brain resulting in deficiencies in minimizing prediction errors during action prediction. As Park and Reuter-Lorenz [22] noted, an efficient task performance relies on an efficient neural circuitry. To the extent that the functionality of these specialized networks declines with age and scaffolding takes place, task performance is likely to get less specific as well. One should also note that this relation presumably depends on additional factors, for example, the connectivity between different brain regions or hemispheres (cf., [28], [102], [103]).

### Involvement of the caudate in action prediction

During the prediction of the figure skating elements compared to movement exercises, a cluster in the caudate extending to the thalamus was more engaged in younger adults compared to older adults. The activation comprised additionally the left PCC for this direction of the contrast. The caudate is connected to various regions in the cerebral cortex, including inferior frontal and inferior parietal regions of the AON [104]–[106]. Activity in the caudate is typically linked to performance monitoring in ambiguous contexts, possibly via representing and updating the value of future actions (i.e., the reward-prediction error; e.g., [107]–[109]). Interestingly, this reward-related recruitment has been found not only in experiential but also in observational instrumental learning tasks [110]. Thus, the caudate appears to be an ideal candidate for neural scaffolding in younger adults during action prediction in conditions of higher difficulty (i.e., lower motor familiarity). In line with this, Schiffer and Schubotz [55] showed that the caudate is involved in prediction errors that are not related to some kind of reward, but violate predictions about which movements should follow after a certain cue in a movement sequence during action observation. The study also reported activation in the PCC for unexpected movement continuations, a region which has been associated with fast visuospatial orientation in unpredictable contexts [111]. Accordingly, PCC activity among younger adults of the present study might have reflected visuospatial monitoring of the more ambiguous figure skating elements.

Our data further show that in figure skating experts compared to non-experts a very similar cluster in the thalamostriatal network was more activated during the prediction of the figure skating elements compared to the movement exercises. Although there was no main effect of action category in the present behavioral data, the study reported by Diersch et al. [8], which used a more fine-grained psychophysical paradigm, showed that the movement exercises were easier to predict than the figure skating elements even for figure skating experts. Due to the small sample size of the experts in the present study, however, this finding needs further confirmation from studies comprising larger sample sizes. The consistency of the results across groups that are in line with previous research still implies that higher prediction errors might not only modulate activity in the AON but also engage the caudate (together with the thalamus), possibly to adjust and optimize less precise predictions that are generated in the AON. As it was demonstrated in the putamen for stimulus-response behaviors, the caudate might similarly modulate information-transfer between visual and motor areas in action-outcome behaviors (cf., [112]). The fact that the caudate was also active together with the hippocampus in older adults during the prediction of the movement exercises emphasizes again its importance during the prediction of actions that are rather ambiguous for the respective observer. Notably, hippocampal activity has been recently linked to the adaptation of stored action representations in younger adults in conditions in which previously encountered action sequences are repeatedly observed in a new, divergent version [56]. Whether the hippocampus might fulfill a similar role in older adults during the prediction of familiar actions poses an important question for future research in order to examine the role of observational learning in older adults in action-related contexts.

## Conclusion

The present study has demonstrated that generating predictive representations of observed actions engages a distributed network in the brain, depending on the characteristics of the observer and the type of observed actions. Based on the predictive coding account, a model was outlined that considers AON activity in relation to the level of motor familiarity in the observer. Moreover, the results underline a role of the caudate during action prediction in ambiguous contexts. In older adults, evidence was found for neural dedifferentiation in relevant areas and engagement of additional regions in line with STAC [22]. Older adults might be considered, metaphorically speaking, as non-experts in previously well-known domains due to internal action representations that become less precise with advancing age. Thus, emphasizing alternative (visual/mnemonic) strategies in training and intervention programs targeted at older adults may provide a promising alternative that supports successful performance in everyday life despite changes in sensorimotor processing.

## Supporting Information

Table S1 **Characteristics of the sample divided by expertise group.**
(PDF)

Table S2 **Effects of each predicted action category compared to baseline collapsed across the whole sample.**
(PDF)

Table S3 **Effects of predicted action category within each age group.**
(PDF)

Table S4 **Effects of predicted action category within each expertise group.**
(PDF)
