# Supplementary material for: Action Prediction in Younger versus Older Adults: Neural Correlates of Motor Familiarity
Source: PLoS One. 2013 May 21;8(5):e64195. doi: 10.1371/journal.pone.0064195 (PMC3660406; doi:10.1371/journal.pone.0064195)
Supplement: Table S1 — Characteristics of the sample divided by expertise group. (PDF) [file pone.0064195.s001.pdf]

Table S1. Characteristics of the sample divided by expertise group.

	**Figure skating experts (n = 10)**	**Non-experts (n = 24)**	***t*(32)**	***p***
Age	35.1 (18.4)	41.5 (20.5)	0.85	0.399
Handedness score	86.4 (10.5)	95.0 (7.59)	2.69	0.011
Years of education	15.7 (3.68)	15.2 (2.99)	0.41	0.686
DSST raw score	76.9 (21.8)	74.4 (16.3)	0.37	0.712
DSST standardized score	11.3 (2.14)	11.0 (3.56)	0.30	0.769
SWT raw score	30.4 (3.10)	33.5 (2.04)	3.50	0.001
SWT standardized score	0.06 (0.42)	0.61 (0.46)	3.26	0.003

Values represent mean scores and standard deviations (parenthesized). See Table 1 for a description of the scores.
