# Supplementary material for: Action Prediction in Younger versus Older Adults: Neural Correlates of Motor Familiarity
Source: PLoS One. 2013 May 21;8(5):e64195. doi: 10.1371/journal.pone.0064195 (PMC3660406; doi:10.1371/journal.pone.0064195)
Supplement: Table S2 — Effects of each predicted action category compared to baseline collapsed across the whole sample. (PDF) [file pone.0064195.s002.pdf]

Table S2. Effects of each predicted action category compared to baseline collapsed across the whole sample.

Anatomical region	Putative functional name	BA	Cluster size	MNI coordinates (mm) *x*	*y*	*z*	*t* value *df* = [1,33]	*p* value (corr.)
(A) Figure skating elements > Baseline								
L Middle Occipital Gyrus	V5/hMT+	19	2101	-45	-73	4	18.71	< 0.001
R Middle Temporal Gyrus	V5/hMT+	19		48	-70	1	13.06	
R Fusiform Gyrus		37		42	-46	-20	11.30	
R Superior Frontal Gyrus	SMA	6	4931	9	5	55	14.67	< 0.001
L Superior Frontal Gyrus	SMA	6		-6	8	52	13.99	
R Middle Frontal Gyrus/R Precentral Gyrus	PMd	6		42	-1	46	13.30	
R Superior Temporal Gyrus	TPJ	22	170	66	-34	16	8.56	0.008
R Midbrain			444	6	-25	-11	7.97	< 0.001
L Midbrain				-3	-25	-11	7.84	
R Putamen/R Globus Pallidum				18	8	1	5.36	
R Superior Parietal Lobule	SPL	7	318	30	-46	46	6.77	< 0.001
R Supramarginal Gyrus	IPL	7/40		36	-34	43	6.28	
R Postcentral Gyrus	S1	2		57	-22	40	3.98	

(B) Movement exercises > Baseline								
L Middle Occipital Gyrus	V5/hMT+	19	641	-45	-73	4	15.77	< 0.001
L Middle Occipital Gyrus	V2/V3	18		-12	-100	7	8.52	
R Superior Frontal Gyrus	SMA	6	7549	9	5	55	13.45	< 0.001
R Middle Temporal Gyrus	V5/hMT+	19		48	-70	-2	13.04	
L Superior Frontal Gyrus	SMA	6		-6	5	52	12.77	
R Midbrain			320	6	-25	-11	7.69	< 0.001
L Midbrain				-6	-25	-5	6.74	

Regions activated during the prediction of figure skating elements (A) and movement exercises (B) compared to baseline. Results are collapsed across the whole sample using a voxel-wise threshold of $p < 0.001$ and a minimum cluster size of 10 voxels. Only clusters are reported that reached cluster-corrected significance of $p < 0.05$, FWE corrected. Up to three local maxima are listed when a cluster has multiple peaks more than 8 mm apart. Abbreviations for brain regions: V5/hMT+, visual area V5/extrastriate visual cortex/middle temporal; SMA, supplementary motor area; PMd, dorsal premotor cortex; TPJ, temporoparietal junction; SPL, superior parietal lobule; IPL, inferior parietal lobule; S1, primary somatosensory area; V2, visual area V2/prestriate visual cortex; V3, visual area V3/extrastriate visual cortex.
