# Supplementary material for: Action Prediction in Younger versus Older Adults: Neural Correlates of Motor Familiarity
Source: PLoS One. 2013 May 21;8(5):e64195. doi: 10.1371/journal.pone.0064195 (PMC3660406; doi:10.1371/journal.pone.0064195)
Supplement: Table S3 — Effects of predicted action category within each age group. (PDF) [file pone.0064195.s003.pdf]

Table S3. Effects of predicted action category within each age group.

				MNI coordinates (mm)			*t* value	*p* value
Anatomical region	Putative functional name	BA	Cluster size	*x*	*y*	*z*		(corr.)
(A) Figure skating elements > Movement exercises								
Younger adults *df* = [1,17]								
R Lingual Gyrus	V2/V3	18	3223	12	-73	-8	10.44	< 0.001
Midline Calcerine Gyrus	V1	17		0	-88	-5	8.86	
R Cuneus	V3	18/19		12	-88	34	8.23	
Older adults *df* = [1,13]								
Midline Calcerine Gyrus	V1	17	1458	0	-91	-8	8.90	< 0.001
R Fusiform Gyrus		19		24	-64	-11	8.58	
R Lingual Gyrus	V1	17		15	-64	1	7.73	
L Medial Orbitofrontal Gyrus	OFC	32	88	-6	35	-8	5.92	0.019
R Medial Orbitofrontal Gyrus	OFC	32		6	38	-8	4.96	
(B) Movement exercises > Figure skating elements								
Younger adults *df* = [1,17]								
R Middle Frontal Gyrus/R Precentral Gyrus	PMd	6	97	33	-7	49	6.67	0.026

R Supramarginal Gyrus	IPL	40	159	54	-28	37	6.35	0.003
R Inferior Parietal Lobule	IPL	40		45	-34	46	4.61	
R Superior Parietal Lobule	SPL	7	214	33	-49	58	6.15	0.001
R Superior Parietal Lobule	SPL	7		21	-58	58	5.22	
R Inferior Temporal Gyrus	V5/hMT+	19	167	48	-73	-5	5.96	0.002
R Middle Temporal Gyrus	MTG	39		45	-61	7	5.37	
R Inferior Frontal Gyrus (pars opercularis)	PMv	44	109	57	11	25	5.77	0.017
L Inferior Parietal Lobule	IPL	40	106	-39	-31	37	4.90	0.019
Older adults *df* = [1,13]								
R Postcentral Gyrus	S1	2	905	36	-37	52	10.49	< 0.001
R Intraparietal Sulcus	IPS	7/40		36	-46	52	9.74	
R Intraparietal Sulcus	IPS	7		33	-67	46	5.97	
L Intraparietal Sulcus	IPS	7/40	660	-39	-43	49	9.00	< 0.001
L Intraparietal Sulcus	IPS	7/2/40		-30	-40	43	8.05	
L Superior Parietal Lobule	SPL	5/2		-30	-49	61	7.14	
L Middle Temporal Gyrus	MTG	39	98	-48	-67	7	8.62	0.012
R Superior Temporal Gyrus	pSTS	22	370	63	-46	13	8.01	< 0.001
R Superior Temporal Gyrus	STG	42		60	-37	16	7.94	

R Middle Temporal Gyrus	pSTS	22		60	-46	4	7.29	
R Inferior Frontal Gyrus (pars opercularis)	PMv	44	322	45	8	16	6.69	< 0.001
R Inferior Frontal Gyrus (pars opercularis)	PMv	44		57	17	28	6.53	
R Precentral Gyrus	PMv	6		48	5	31	5.57	
L Superior Frontal Gyrus	SMA	6	158	-9	-1	52	6.65	0.001
L Superior Frontal Gyrus	PMd	6		-24	-10	55	5.83	
L Superior Frontal Gyrus	SMA	6		-9	-19	58	5.01	

Regions activated during the prediction of figure skating elements compared to movement exercises (A) and vice versa (B) in younger and older adults while controlling for figure skating expertise. Results were calculated using a voxel-wise threshold of $p < 0.001$ and a minimum cluster size of 10 voxels. Only clusters are reported that reached cluster-corrected significance of $p < 0.05$, FWE corrected. Up to three local maxima are listed when a cluster has multiple peaks more than 8 mm apart. Abbreviations for brain regions: V2, visual area V2/prestriate visual cortex; V3, visual area V3/extrastriate visual cortex; V1, visual area V1/striate visual cortex; OFC, orbitofrontal cortex; PMd, dorsal premotor cortex; IPL, inferior parietal lobule; SPL, superior parietal lobule; V5/hMT+, visual area V5/extrastriate visual cortex/middle temporal; MTG, middle temporal gyrus; PMv, ventral premotor cortex; S1, primary somatosensory area; IPS, intraparietal sulcus; pSTS, posterior superior temporal sulcus; STG, superior temporal gyrus; SMA, supplementary motor area.
