# Supplementary material for: Action Prediction in Younger versus Older Adults: Neural Correlates of Motor Familiarity
Source: PLoS One. 2013 May 21;8(5):e64195. doi: 10.1371/journal.pone.0064195 (PMC3660406; doi:10.1371/journal.pone.0064195)
Supplement: Table S4 — Effects of predicted action category within each expertise group. (PDF) [file pone.0064195.s004.pdf]

Table S4. Effects of predicted action category within each expertise group.

				MNI coordinates (mm)			*t* value	*p* value
Anatomical region	Putative functional name	BA	Cluster size	*x*	*y*	*z*		(corr.)
(A) Figure skating elements > Movement exercises								
Figure skating experts *df* = [1,8]								
R Cuneus	V1/V2	17/18	519	6	-88	16	17.37	< 0.001
R Calcerine Gyrus	V1/V2	17/18		3	-85	-5	14.98	
R Lingual Gyrus	V2	18		9	-79	-8	12.50	
L Caudate			188	-9	-7	16	8.76	< 0.001
L Thalamus				-6	-7	1	8.33	
L Putamen				-24	-16	13	8.15	
R Thalamus			63	9	-7	-2	7.02	0.038
R Thalamus				9	-1	4	6.06	
R Caudate				9	5	10	5.60	
Non-experts *df* = [1,22]								
R Parahippocampal/Lingual Gyrus		18	2944	27	-49	-2	9.67	< 0.001
R Lingual Gyrus	V3	18/19		15	-70	-8	9.37	

Midline Calcerine Gyrus	V1	17		0	-88	-5	8.86	
L Superior Frontal Gyrus	SFG	8	92	-12	41	52	5.60	0.033
L Superior Frontal Sulcus	SFS	8/9		-24	29	55	4.99	
L Superior Frontal Gyrus	SFG	8		-12	32	58	4.83	
(B) Movement exercises > Figure skating elements								
Figure skating experts *df* = [1,8]			------- No suprathreshold activations -------					
Non-experts *df* = [1,22]								
L Precuneus		7	1050	-9	-58	58	7.95	< 0.001
L Intraparietal Sulcus	IPS	7/40		-42	-40	49	7.10	
L Superior Frontal Gyrus	PMd	6		-24	-10	55	6.74	
R Supramarginal Gyrus	IPL	2/40	1043	48	-34	43	7.61	< 0.001
R Superior Parietal Lobule	SPL	7		36	-49	58	7.09	
R Postcentral Gyrus	S1	2		54	-25	43	6.68	
R Inferior Frontal Gyrus (pars opercularis)	PMv	44	714	51	8	28	7.49	< 0.001
R Middle Frontal Gyrus	PMd	6		36	-7	52	7.27	
R Superior Frontal Sulcus	SFS	6/8		24	11	43	4.28	
R Superior Temporal Gyrus	TPJ/STG	22/42	442	60	-34	16	6.76	< 0.001
R Middle Temporal Gyrus	MTG	37/39		57	-61	1	6.41	

R Middle Temporal Gyrus	pSTS	22		57	-40	7	5.96	
L Middle Occipital Gyrus	V5/hMT+	39	139	-45	-70	4	5.44	0.007
L Middle Occipital Gyrus	V5/hMT+	19		-45	-76	-2	5.32	
L Precentral Gyrus	PMv	6/44	107	-48	5	19	4.93	0.019
L Precentral Gyrus	PMv	6		-51	5	37	4.76	
L Inferior Frontal Gyrus (pars opercularis)	PMv	44		-54	8	10	3.94	

Regions activated during the prediction of figure skating elements compared to movement exercises (A) and vice versa (B) in figure skating experts and non-experts while controlling for age group. Results were calculated using a voxel-wise threshold of $p < 0.001$ and a minimum cluster size of 10 voxels. Only clusters are reported that reached cluster-corrected significance of $p < 0.05$, FWE corrected. Up to three local maxima are listed when a cluster has multiple peaks more than 8 mm apart. Abbreviations for brain regions: V1, visual area V1/striate visual cortex; V2, visual area V2/prestriate visual cortex; V3, visual area V3/extrastriate visual cortex; SFG, superior frontal gyrus; SFS, superior frontal sulcus; IPS, intraparietal sulcus; PMd, dorsal premotor cortex; IPL, inferior parietal lobule; SPL, superior parietal lobule; S1, primary somatosensory area; PMv, ventral premotor cortex; TPJ, temporoparietal junction; STG, superior temporal gyrus; MTG, middle temporal gyrus; pSTS, posterior superior temporal sulcus; V5/hMT+, visual area V5/extrastriate visual cortex/middle temporal.
